# A Point-of-Care test for facing the burden of undiagnosed celiac disease in the Mediterranean area: a pragmatic design study

**DOI:** 10.1186/s12876-014-0219-5

**Published:** 2014-12-18

**Authors:** Stefano Costa, Luca Astarita, Mongi Ben-Hariz, Giovanni Currò, Jernej Dolinsek, Aydan Kansu, Giuseppe Magazzu’, Stefania Marvaso, Dusanka Micetic-Turku, Salvatore Pellegrino, Giuseppe Primavera, Pasqualino Rossi, Andrea Smarrazzo, Francesca Tucci, Carmela Arcidiaco, Luigi Greco

**Affiliations:** Celiac Regional Center, Pediatric Gastroenterology and Cystic Fibrosis Unit, University of Messina, Via Consolare Valeria 1, 98125 Messina, Italy; European Laboratory for Food Induced Diseases, University of Naples Federico II, Naples, Italy; Pediatric Unit, Mongi SLIM’s Hospital of Tunis, Marsa, Tunisia; University Medical Centre Pediatric Department, Ljubljanska, Maribor, Slovenia; Faculty of Medicine, Department of Pediatric Gastroenterology, Ankara University, Ankara, Turkey; National Health System, Azienda Sanitaria Locale 6, Associazione Culturale Pediatri, Palermo, Italy; Directorate General for European and International Relations, Ministry of Health, Rome, Italy; Department of Translational Medical Science, Section of Pediatrics, University of Naples Federico II, Naples, Italy

**Keywords:** Celiac disease, Diagnosis, Mediterranean area, Point-of-care test, Rapid test

## Abstract

**Background:**

We aimed at assessing the factors that can influence results of the dissemination of an already validated, new generation commercial Point-of-Care Test (POCT) for detecting celiac disease (CD), in the Mediterranean area, when used in settings where it was designed to be administered, especially in countries with poor resources.

**Methods:**

Pragmatic study design. Family pediatricians at their offices in Italy, nurses and pediatricians in Slovenia and Turkey at pediatricians’, schools and university primary care centers looked for CD in 3,559 (1-14 yrs), 1,480 (14-23 yrs) and 771 (1-18 yrs) asymptomatic subjects, respectively. A new generation POCT detecting IgA-tissue antitransglutaminase antibodies and IgA deficiency in a finger-tip blood drop was used. Subjects who tested positive and those suspected of having CD were referred to a Celiac Centre to undergo further investigations in order to confirm CD diagnosis. POCT Positive Predictive Value (PPV) at tertiary care (with Negative Predictive Value) and in primary care settings, and POCT and CD rates per thousand in primary care were estimated.

**Results:**

At tertiary care setting, PPV of the POCT and 95% CI were 89.5 (81.3-94.3) and 90 (56-98.5) with Negative Predictive Value 98.5 (94.2-99.6) and 98.7% (92-99.8) in children and adults, respectively. In primary care settings of different countries where POCT was performed by a different number of personnel, PPV ranged from 16 to 33% and the CD and POCT rates per thousand ranged from 4.77 to 1.3 and from 31.18 to 2.59, respectively.

**Conclusions:**

Interpretation of POCT results by different personnel may influence the performance of POC but dissemination of POCT is an urgent priority to be implemented among people of countries with limited resources, such as rural populations and school children.

## Background

Celiac Disease (CD) is a systemic immune-mediated disorder triggered by dietary gluten in genetically susceptible persons and is characterized by a broad range of clinical presentations and variable damage to the small-intestinal mucosa [1].

The active disease is characterized by gluten-dependent auto-antibodies against endomysium and, more precisely, protein type 2 (‘tissue’) transglutaminase, the celiac autoantigen anchored to endomysial collagen by fibronectin [2,3].

Detection of these auto-antibodies in serum represents a valuable tool for identifying new celiac patients presenting with only mild gastro-intestinal symptoms, non-specific general complaints or extra-intestinal manifestations, or for screening asymptomatic subjects [4,5]. The prevalence of CD in various populations is around 1% [6-8].

The burden of unrecognized CD in the Mediterranean area has been estimated in terms of morbidity costs and mortality [9]. The projected number of CD diagnoses in 2020 is 5 million cases (1 million celiac children), with a relative increase of 11% compared to 2010. The estimated standardized medical costs for symptomatic celiac patients during the delay between symptom onset and diagnosis (mean - 6 years for adults, 2 years for children) will be about €4 billion (€387 million for children) over the next 10 years. A delay in diagnosis is expected to increase morbidity, in terms of nutritional deficiencies and, especially, of gluten related autoimmune diseases and mortality: about 600,000 celiac patients will die in the next 10 years, with an excess of 44.4% versus age- and sex-matched controls. In order to face this non-communicable epidemic, it is necessary to have accurate tests available.

IgA anti tissue transglutaminase antibodies (IgA-tTG) from commercial enzyme-linked immunosorbent assays (ELISA) utilized for CD testing require a serum sample, need equipped laboratories and are too expensive for countries with poor resources, such as most of those in the Mediterranean area, where patients and families are not able to reach referral centers or centralized laboratories located far away.

Due to the necessity of drawing blood in a CD screening study, 30% of parents of children initially selected refused blood sampling [10].

There is thus the need for quick, easy-to-perform, low-cost and widely accessible celiac antibody tests which can be carried out at the Point-of-Care (POC) in countries with poor resources.

For this reason, a rapid test for detecting CD was developed more than 20 years ago [11].

Since this first home-made assay, which was not applied in clinical practice, several articles have recently been published on new commercial rapid tests [12-31], regarding also an assay which detects anti-deamidated gliadin peptides [31].

Among these, a POC test (POCT) has already been validated, mostly in tertiary centers [13,15-18,20,22,23,26,27] and in primary care settings [14,19,21,24,25]. However, both the first [14,19,21] and the new generation [24,25] POCT have been administered by personnel, generally one person, dedicated to performing the test [14,24] or by the same researchers [21,25]. The exception is a study performed in Hungary where 120 district nurses screened 6-year old children for CD with a first generation POCT and demonstrated 100% specificity, but a lower sensitivity of the test [19]. Therefore, it is important to assess if a test conceived for point-of-care utilization can maintain its accuracy in settings where it will be disseminated.

The Medicel network (www.medicel.unina.it/) is a project supported by the Italian Ministry of Health, Direction of International Affairs and comprises 16 countries with different resources and facilities (and limitations) for diagnosing CD [32], ranging from those that have tertiary level CD centers and family doctors to those in which, apart from lack of facilities and personnel, there are economic restraints for referring patients suspected of having celiac disease to Health Centers.

We aimed at assessing the factors that can influence results of the dissemination of an already validated new generation commercial POCT for detecting CD, in the Mediterranean area, when it is used in the settings where it was designed to be administered.

## Methods

We adopted a pragmatic design in which potential bias has been assessed. Awareness of the potential biases and its implications allows us to discuss possible solutions and overcome such bias.

Celiac centers from three countries, Italy, Slovenia and Turkey, participated in the study. Ethical institutional review boards at each collection site approved the study in the 3 centers participating in the study: in Italy, at the University Hospital, Messina; in Slovenia at the University Medical Centre Ljubljanska, Maribor; in Turkey, at the Faculty of Medicine, Ankara University, Ankara. Written informed consent for participation in the study was obtained from participants or, where participants were children, a parent or guardian.

In Table 1, demographic data of enrolled subjects and information on setting and personnel performing the test in the various countries participating in the study are shown.

**Table 1:** **Setting of the study, personnel performing the test and demographic data of enrolled subjects**

**Country**	**Italy**	**Slovenia**	**Turkey**
**Setting**	A) Primary care: Family pediatrician’s office	D) Primary care – screening of secondary school students – mostly in rural area of NE Slovenia	Primary care: Pediatrician’s office
	B) Celiac centre pediatric patients	E) Students from Medical Faculty and Faculty of Health Sciences University of Maribor - screening	
	C) Celiac centre adult patients		
**Personnel performing POCT**	A) Family pediatricians	D) Trained nurse, student and pediatrician, pediatric gastroenterologist – as supervisor	Nurse and pediatrician
	B) Physician and biologist	E) Trained nurse, trained students, pediatric gastroenterologist – as supervisor	
	C) Physician and biologist		
**No. subjects tested (age range)**	A) 3,559 (1-14 yrs) asymptomatic children	A) 1,000 (14-18y) secondary school students	771 (1-18 years) asymptomatic children at school (666) and at a primary care pediatrician’s office (105)
	B) 206 (1-18 yrs) children for suspected CD	B) 480 (18-23y) University students	
	C) 85 (>18 yrs) adults for suspected CD		

The “new generation” Biocard™ celiac test (AniBiotech®, Vantaa, Finland), based on an immunochromatographic technique, was used. This technique detects IgA-tTG in whole blood. The procedure was performed as previously described [16]: with a drop of whole blood taken by finger prick, with bands visually detected after 5 minutes but no later than 10 minutes as recommended by the manufacturer’s instructions.

Moreover, the “new generation” rapid test contains a second line (control line) with an antihuman IgA antibody. The test result is positive if both lines are seen, negative if only the control line forms. In the case of IgA deficiency, no line is visible on the test.

In Italy, the study involved 39 family pediatricians in Sicily. At their offices, all consecutive asymptomatic children were offered the POCT and those who tested positive or were found to be IgA negative on Biocard testing were referred to a Celiac Center (Group A in Table 1). Moreover, all consecutive children (Group B) and adults (Group C) referred for symptoms suggestive of CD by other physicians, or because they were first degree relatives of celiac patients, were enrolled at the referral Centre where they underwent both POCT, performed by a biologist or a physician, and conventional celiac serology determination.

In Slovenia, participants spontaneously underwent POCT from 6 secondary schools and 1 Medical Faculty after public invitation. POCT was performed by one pediatrician, one trained nurse and 2 trained students with a pediatric Gastroenterologist as supervisor. Subjects who tested positive or with no line were sent to the Referral Celiac Center to undergo conventional celiac serology.

In Turkey, asymptomatic children at school and at a primary care pediatrician’s office underwent POCT performed by trained pediatricians and nurses. All children who tested positive or were found to be IgA negative on Biocard testing were referred to a tertiary level center to undergo celiac conventional serology.

In all the Centers, conventional IgA-tTG assays were performed with one of the kits recommended by ESPGHAN [33].

Subjects who had positive serology underwent upper gastrointestinal endoscopy with duodenal biopsies. Histology was classified according to the Marsh criteria modified by Oberhuber [34]. All patients definitively diagnosed as having celiac disease were consequently given a gluten-free diet.

Sensitivity, specificity, Positive and Negative Predictive Value (PPV and NPV, respectively) and positive and negative likelihood ratio were calculated for POCT performed in patients referred to the Celiac Center in Italy. Intestinal biopsy was performed in all subjects in whom one of the tests, including POCT, was positive at the Center, and Marsh type 2 (in children) or 3 at histology was considered the gold standard of CD diagnosis.

Positive Predictive Value of POCT and rate per thousand positive POCT and CD were calculated for subjects in whom POCT was performed by different persons in other settings. In these cases, intestinal biopsy was performed if one of the conventional celiac serology tests was positive and histology was considered the gold standard. False positive POCTs were considered subjects with normal conventional celiac serology, as, in these asymptomatic children, performing intestinal biopsy was judged unethical.

At tertiary centers, laboratory personnel were blinded to POCT results while assaying celiac conventional serology.

## Results

In Table 2, the performance of POCT at the Italian Celiac Center is shown.

**Table 2:** **Diagnostic accuracy of POCT at the Italian Celiac Centre**

**Subjects**	**No. Tested**	**No. positive**	**No. negative**	**Sensitivity %**	**Specificity %**	**PPV %**	**NPV %**	**LR +**	**LR -**
		**(No.CD found)**	**(No.CD found)**						
Paediatric	206	76	130	97.1	94.1	89.5	98.5	16.5	0.03
		(68)	(2)	(93.1-100)	(90.1-98.1)	(81.3-94.3)	(94.2-99.6)	(8.4-32.39)	(0.01-0.12)
Adult	85	10	75	90.0	98.7	90.0	98.7	67.5	0.10
		(9)	(1)	(70.4-100)	(96.1-100)	(56.0-98.5)	(92.0-99.8)	(9.5-478)	(0.02-0.65)

(95% CI).

PPV: Positive Predictive Value.

NPV: Negative Predictive Value.

LR: Likelihood ratio.

In Table 3, POC PPV and rates per thousand for POCT and CD are shown.

**Table 3:** **Rate of positive Point-of-care Test and Celiac disease found according to personnel performing the test**

					**Rate per thousand**	
**Country**	**Persons (and No.) performing test**	**Subjects tested**	**POCT + (PPV)**	**CD**	**POCT +**	**CD**
ITALY	Family (39) pediatricians	3559	111 (16%)	17	31.18	4.77
SLOVENIA	Nurse (1)	1480	18 (33%)	7	12.16	4.72
	Student (2)					
	Pediatrician (1)					
TURKEY	Pediatrician (2) Nurse (1)	771	2* (N.A.)	1	2.59	1.30

*1 patient was not referred to Center to undergo conventional serology and histology and thus PPV was Not Available (NA).

CD: Celiac Disease.

POCT: Point-of-Care Test.

The lowest PPV was found in Italy and Slovenia but in these countries POCT yielded a high rate of positive cases and of CD.

All false positive cases of POCT (84%) performed by pediatricians in primary care in Italy were negative at the POCT performed at the Center by a well experienced biologist or physician. In Slovenia, there were 67% false positive cases while it was not possible to estimate the number in Turkey as, out of 2 children with positive POCT, one refused referral to the Celiac Center.

**Figure 1:**
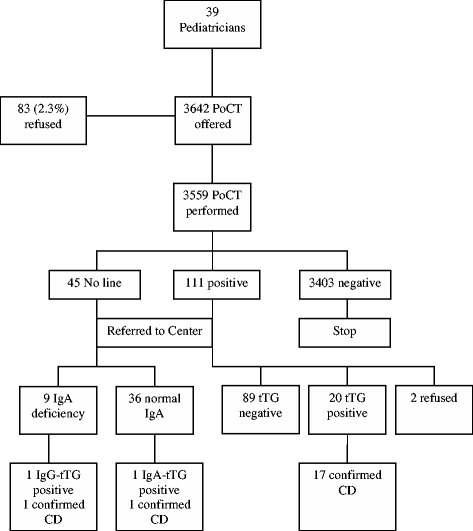
**Results in primary care in Italy.** Abbreviations: PoCT= Point-of-Care Test; tTG = anti-tissue transglutaminase antibodies.

**Figure 2:**
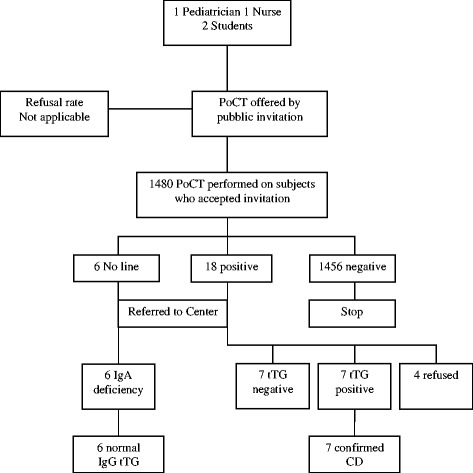
**Results in primary care in Slovenia.** Abbreviations: PoCT= Point-of-Care Test; tTG = anti-tissue transglutaminase antibodies.

**Figure 3:**
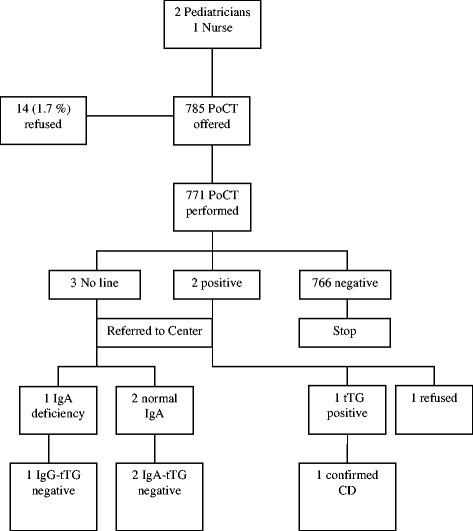
**Results in primary care in Turkey.** Abbreviations: PoCT= Point-of-Care Test; tTG = anti-tissue transglutaminase antibodies.

Diagrams of results obtained in Italy, Slovenia and Turkey are shown in Figures 1, 2 and 3, respectively.

In Italy (Figure 1), out of 45 cases who had no line visible on the test, 37 had normal IgA at the Center. In one of these, IgA-tTG was high and histology confirmed CD. In another case with confirmed IgA deficiency, IgG-tTG was high and histology confirmed CD. Out of 20 children with positive tTG confirmed at the Center, 3 had low titers of tTG (<4 X upper limit of normal) and absent antiendomysial antibodies. Following requests by the families, the children have not yet undergone duodenal biopsy and are in follow up with a gluten containing diet. The lowest ELISA value among the 17 children with POCT positive in whom CD diagnosis was confirmed was about four fold the cut-off.

The refusal rate of parents to allow their children to undergo POCT in the pediatrician’s office was 2.3%.

In Slovenia (Figure 2) the refusal rate was not estimable due to the spontaneous participation of subjects after public invitation.

In Turkey (Figure 3) the refusal rate was 1.7%. One family refused duodenal biopsy in one out of the 2 children who resulted tTG positive and, therefore, it was not possible to estimate CD rate per thousand (Table 3).

## Discussion

Our pragmatic study was aimed at getting information to then disseminate a well validated, cheap and rapid test for CD that does not require an equipped laboratory, above all in some Mediterranean countries where most of the patients do not have enough resources to reach a referral centre.

The ideal test should be cheap and feasible, with very high sensitivity and specificity.

Considering that the POCT used in our study has previously been validated, and that its high diagnostic accuracy has been confirmed in the present study at the tertiary level center in Italy, variations in PPV of the new generation POCT do not depend on the test but rather, apart from the prevalence of higher CD in tertiary care centers than in community settings, on the operator and interpretation of test. It is of interest that in our study, the lowest PPV was obtained in settings where the POCT was performed by more than one operator, such as primary care pediatricians in their office or personnel who are not completely dedicated to performing diagnostic procedures. In Slovenia, where all the POCTs were supervised by a skilled pediatric gastroenterologist with long experience with these tests, a lower level of false positive was obtained in comparison with that observed among primary care pediatricians. In a screening study performed in Tunisia, using the same new generation POCT [25], the interpretation of faint test lines explained different PPVs obtained. Moreover, in a previous study performed in Hungary by 120 non-trained district nurses [19], in which each one performed a median of 18 first generation POC tests, specificity was 100% but sensitivity of the test was low. These results may be due, as the authors suggested, to the lower sensitivity obtained on site by the nurses interpreting faint test lines as negative results. In our study, differences between positive tests and faint test lines were not recorded. Therefore, we can only speculate that personnel in primary care also referred subjects to the Center with faint test lines who afterwards tested negative at conventional serology, as shown in a previous study [25]. On the other hand, it was reported that doubtful results with faint lines of positivity have actually been observed at the end of the reading time recommended by the manufacturer (9–10 minutes). Further investigation by serology revealed that all of these “doubtful” results were false positive [25]. Unfortunately, we did not record the test reading exact time and participants were asked to read the test within 10 minutes as recommended by the manufacturer.

In countries with poor resources, it would be advisable for this POCT to be performed by a minimum number of trained people, in order to minimize costs induced by false positive cases.

Two previously published studies which were performed in two centers (Greece and Tunisia) from countries participating in the Medicel network, using the same POCT, obtained the highest PPV (100%) [24,25].

In Greece [24], properly trained nonmedical staff performed the POCT on toddlers attending nursing school.

In Tunisia [25], screening for CD by POCT was performed by a team of 3 doctors and a nurse previously trained in reading the test. Among the children who tested negative with POCT, a small sample consisting of the first 54 were called in for conventional testing in order to assess NPV of the test. NPV, although estimated in a small sample, was 100%.

In the present study, NPV of POCT was assessed in a tertiary level Centre in a population with high and moderate prevalence of CD, such as that represented by patients referred with the suspicion of CD and first degree relatives, respectively. POCT at the tertiary centre, and in a population with the above cited prevalence of CD, had a very high NPV but this must be assessed in settings other than a Celiac centre and in populations with low prevalence of CD. NPV was previously reported lower in a study performed in Libyan children who participated in a screening study but the results were based on a first generation POCT [21]. However, while waiting for further studies aimed at assessing NPV by assaying conventional CD serology in patients with negative POCT, we believe that it is cost-effective to start huge case finding and, above all, screening strategies [35,36] based on POCT in order to face the epidemic of CD and decrease morbidity, mortality and costs in the Mediterranean area [9,32]. These costs, even in the worst possible scenario, allow us to save money due to the excessive costs of patients with undiagnosed CD, keeping in mind that the so-called active case-finding may fail to identify the majority of them [35,36].

## Conclusions

Our study suggests that factors that can influence the results of a POCT dissemination in the Mediterranean area, apart from the prevalence of CD in different settings where it is applied, include interpretation of results by appropriately trained personnel performing the test. More studies are needed to decide the correct time limit for test and interpretation of faint lines.

We believe that the populations amongst which the dissemination of POCT would be of the greatest benefit and where it is most "urgent" to implement are in countries with limited resources, above all rural populations where most of the people are not able to reach the referral centres.

## Abbreviations

CD: Celiac disease
IgA-tTG: IgA-tissue antitransglutaminase antibodies
POCT: Point-of-Care Test
